# Willingness to Participate in Alcohol Prevention Interventions Targeting Risky Drinking Employees. The WIRUS Project

**DOI:** 10.3389/fpubh.2021.692605

**Published:** 2021-06-25

**Authors:** Mikkel Magnus Thørrisen, Tore Bonsaksen, Jens Christoffer Skogen, Lisebet Skeie Skarpaas, Aleksandra Sevic, Willem van Mechelen, Randi Wågø Aas

**Affiliations:** ^1^Department of Occupational Therapy, Prosthetics and Orthotics, Faculty of Health Sciences, OsloMet – Oslo Metropolitan University, Oslo, Norway; ^2^Department of Public Health, Faculty of Health Sciences, University of Stavanger, Stavanger, Norway; ^3^Department of Health and Nursing Sciences, Faculty of Social and Health Sciences, Inland Norway University of Applied Sciences, Elverum, Norway; ^4^Faculty of Health Sciences, VID Specialized University, Sandnes, Norway; ^5^Department of Health Promotion, Norwegian Institute of Public Health, Sandnes, Norway; ^6^Center for Alcohol & Drug Research, Stavanger University Hospital, Stavanger, Norway; ^7^Department of Public and Occupational Health, Amsterdam Public Health Research Institute, Amsterdam University Medical Centers (Location VUmc), Amsterdam, Netherlands; ^8^School of Human Movement and Nutrition Sciences, Faculty of Health and Behavioral Sciences, University of Queensland, Brisbane, QLD, Australia; ^9^Division of Exercise and Sports Medicine, Department of Human Biology, Faculty of Health Sciences, University of Cape Town, Cape Town, South Africa; ^10^School of Public Health, Physiotherapy and Population Sciences, University College Dublin, Dublin, Ireland

**Keywords:** alcohol, brief interventions, employees, occupational health services, Workplace Interventions, reach, RE-AIM, Sick leave

## Abstract

**Background:** The extent to which eligible individuals in a target population are willing to participate in interventions is important when evaluating the efficacy of public health interventions.

**Objectives:** As part of a process evaluation of an ongoing randomized controlled trial, this study aimed to identify the proportion of risky drinkers who were willing to participate in an alcohol prevention intervention in an occupational health setting, and correlates for such willingness.

**Methods:** Risky drinking employees from 22 companies in Norway were identified through an alcohol screening survey. Risky drinkers' (*N* = 779) willingness to complete a health examination and to be randomized into an alcohol prevention intervention (digital or face-to-face intervention, or control) was recorded by personnel from occupational health services. The proportion of employees who were willing to participate was assessed on 31 potential correlates (sociodemographic, alcohol-related, work-related, and lifestyle/daily activity). Adjusted (multiple logistic regression) analyses were utilized to explore associations between potential correlates and willingness to participate.

**Results:** Altogether, 38.1% of employees were willing to participate in prevention interventions. In the adjusted analysis, only 5 out of 31 potential correlates were significantly associated with willingness to participate. Managers were more than twice as willing to participate than workers (OR = 2.17, *p* < 0.01). Willing employees had less workplace decision latitude (perceived control over workplace decisions and less possibility of utilizing personal skills in the job) (OR = 0.62, *p* < 0.05), and were more overcommitted with exorbitant work ambition and need for approval (OR = 1.49, *p* < 0.05). Willing employees had to some extent less alcohol-related impaired work performance (presenteeism, OR = 0.78, *p* < 0.05), and they spent less time on care activities (OR = 0.84, *p* < 0.05).

**Conclusions:** Reaching four out of ten with risky drinking habits for prevention interventions strengthens the rationale for targeting this public health problem in occupational health care settings. In particular, this study suggests the importance of ensuring secure commitment among workers, who were less willing til participate than managers. Nevertheless, tailoring recruitment and implementation strategies based on easily identifiable correlates may be onerous.

## Introduction

A large body of evidence has linked harmful alcohol consumption to detrimental health outcomes (1, 2), and reducing such consumption has been identified as a keystone in sustainable development of health (3, 4). Alcohol is the most used and misused psychoactive substance in the workforce (5). A considerable proportion of employees (one to three out of ten) can be characterized as risky drinkers, who may benefit from alcohol prevention interventions (6). Risky drinking is defined as a drinking pattern that increases the risk of medical, social, legal, occupational, domestic and economic problems, according to the World Health Organization (7). The majority of risky drinkers are part of the active workforce (8), and primary research as well as systematic reviews have demonstrated that employees' alcohol consumption is associated with sickness absence (9–14), as well as with presenteeism (impaired on-the-job performance due to health impairments, e.g., hang over episodes) (15–19). According to estimates from 2006, alcohol-related absenteeism alone was identified to carry an annual economic burden of 30–65 billion U.S. dollars on a global scale (20).

Studies have demonstrated that brief alcohol prevention interventions may provide favorable effects. Such interventions could be face-to-face consultations with health care professionals (21–23), as well as digital platform interventions (24, 25). However, favorable public health impact of prevention efforts are not solely a matter of efficacy and effectiveness, and several authors have emphasized that alcohol prevention interventions may be challenging to implement in practice (26–28). Within the RE-AIM framework (29), the impact of interventions is considered a function of five factors: reach, efficacy, adoption, implementation, and maintenance. Reach represents an individual-level measure of participation, and is generally defined as the absolute number, proportion, and representativeness of eligible individuals participating in the intervention (29, 30).

Reaching risky drinkers may be challenging, insofar that individuals with issues relating to alcohol consumption may be particularly reluctant to seek and receive help (31). Seeking and receiving help for stigmatized conditions or problems (e.g., in terms of participating in an alcohol prevention intervention) may challenge the individual's self-view (32). This may to a greater extent apply to interventions that require face-to-face interaction compared to eHealth interventions that offer anonymity on a digital platform. Moreover, interventions targeting risky drinking may be conceptualized as secondary or selective prevention measures, i.e., measures aimed at individuals at risk of experiencing an undesirable end-state by being a member of an at-risk subgroup of the population, rather than individuals who have already experienced such a state (e.g., alcohol dependence) (33, 34). Hence, risky drinkers may not have experienced any alcohol-related problems and may thus be reluctant to perceive their alcohol use as problematic. However, inadequately reaching risky drinkers for alcohol prevention may entail missed opportunities for the individual, as well as for society.

In a study of emergency department patients in Sweden (35), a computerized alcohol intervention reached 41% of the target population, which according to the authors represented an acceptable reach. Patients willing to participate appeared similar to those who were unwilling. There were no significant differences based on variables such as gender, educational attainment, occupation, and alcohol consumption. Participants were, however, significantly younger than non-participants, which may to some extent be due to younger individuals being more comfortable and familiar with the digital format than older individuals. A German study of risky drinking general hospital inpatients at four medical departments (36) found an overall participation rate of 81%. The participation rate was slightly higher for a digital intervention with computer-generated individualized feedback than for a face-to-face intervention based on motivational interviewing (digital: 387 of 388 received allocated intervention; face-to-face: 354 of 367 received allocated intervention). In line with the Swedish study, participants and non-participants in the German study did not differ on variables such as gender, employment status, and alcohol consumption. Similar results have been found among American trauma patients eligible for a brief face-to-face intervention administered by health care personnel (37). A study of a general population sample in Germany (visitors at a municipal registry office, i.e., a public authority for registration, passport and vehicle administration issues) (38) did, however, report that low-risk drinkers had higher odds of participating in a digital intervention with computer-generated feedback than risky drinkers. Sociodemographic variables, smoking status, fruit and vegetable intake, physical activity, body mass index, and self-reported health did not differ between participants and non-participants, and participation rate was estimated to 67%.

Research on reach for alcohol interventions is quite sparse and somewhat inconsistent. Moreover, research on reach in employee populations is even more dearth. As the majority of adults are employed and spend considerable time at work (39), and since the majority of risky drinkers in society are part of the active workforce (8), the workplace setting is considered a serviceable arena for alcohol prevention. Several authors have advocated that the occupational health services (OHS) should be placed in a more active role in alcohol prevention targeting employees (40–42). To what extent employee populations may be reached for alcohol prevention, and to what extent employees who are willing to participate are similar to those who are unwilling, remain largely unanswered questions.

Thus, this study aimed (i) to assess the proportion of risky drinking employees willing to participate in an alcohol intervention in an occupational health setting, and (ii) to identify correlates for such willingness, based on the exploration of a wide range of variables. The purpose of the study was to generate knowledge enabling better recruitment and implementation strategies for reaching risky drinkers for alcohol prevention in occupational health care settings.

## Materials and Methods

### Design, Setting, and Procedure

This prospective study was conducted in a heterogeneous sample of 779 employees from 22 large companies in Norway as part of a process evaluation of an ongoing randomized controlled trial in the WIRUS project (Workplace Interventions preventing Risky alcohol Use and Sick leave). For the process evaluation, the RE-AIM concepts were applied—where reach is one of the evaluation categories. To conduct the process evaluation, a standardized questionnaire was filled out by OHS personnel.

First, employees were invited to participate in a digital alcohol screening survey. Second, employees who were classified as risky drinkers (based on their screening responses) were invited by their OHS to attend a health examination, where they were randomized into an alcohol prevention intervention (or control) condition. During the health examination, OHS personnel recorded whether or not employees consented to be randomized. For this study, consent to randomization defined willingness to participate in an alcohol prevention interventions.

### Data Collection and Sample

**Figure 1:**
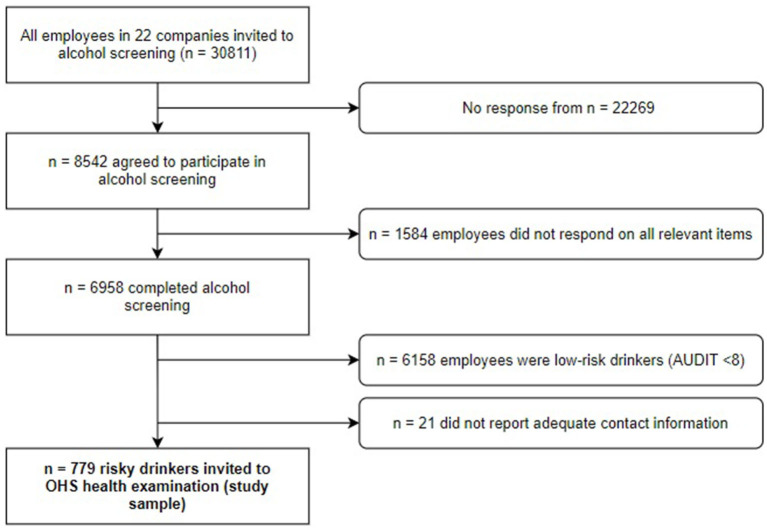
Flow chart depicting the process of participant recruitment. AUDIT, Alcohol Use Disorders Identification Test; OHS, occupational health services.

In collaboration with the addiction competence environment KoRus Stavanger and the University of Stavanger, companies were recruited through three OHS in Norway. Twenty-two companies agreed to participate and provided e-mail addresses for all their employees. Included companies represented a variety of sectors, work divisions and geographical locations. The alcohol screening survey was distributed to all employees in the 22 companies (*n* = 30,811). A total of 8,542 employees consented to participate (response rate = 27.7%), and 6 958 completed the alcohol screening by responding on all relevant items. Based on having a sum score of eight or higher on the Alcohol Use Disorders Identification Test (AUDIT) (7, 43), 800 risky drinkers were identified (11.5% of employees who completed the alcohol screening). Sample characteristics of the screening study are described in more detail elsewhere (6, 16, 44–48). Twenty-one risky drinkers failed to report adequate contact information. Hence, 779 risky drinking employees were contacted by OHS and included in this study. The recruitment process for this study is depicted in Figure 1.

Risky drinkers (*N* = 779) were invited to a general health examination by the OHS and were informed about further potential participation in an alcohol prevention intervention. Invitations were made by e-mail and telephone, based on contact information provided by employees in the screening survey. Up to two reminders were sent in instances where employees did not respond to the OHS' initial invitation. In most cases, attending the health examination involved leaving the workplace and traveling some distance to the OHS facilities. Date and time for the examination were scheduled in concert between the employee and OHS.

The health examination lasted for ~30 min. After having completed the examination, employees were informed about the randomized controlled trial and asked whether they were willing to be randomized into one out of three groups: (i) a face-to-face brief intervention based on motivational interviewing plus an alcohol information booklet; (ii) an eHealth intervention delivered on a digital platform, plus an alcohol information booklet; or (iii) a control condition where they received only the alcohol information booklet. The face-to-face intervention consisted of two motivational interviewing sessions with OHS personnel (49). The digital eHealth intervention started with an alcohol screening and feedback component, followed by an intensive self-help program consisting of 62 online sessions, distributed across a period of 6 months (50, 51). The alcohol information booklet contained general information about physiological effects of alcohol intake (52).

Individual-level criteria for being included in the study were the following: (i) age 16–72; (ii) status as employee (blue, white or pink collar worker, or manager, i.e., salaried person); (iii) employed in a company served by an OHS unit enrolled in the WIRUS project, regardless of sector, work division or geographical region; (iv) basic understanding of the Norwegian language; and (v) completed the screening survey and scored eight or higher on the AUDIT scale (i.e., being classified as a risky drinker). Data were collected between 2014 and 2020.

The mean age for included employees was 40.3 years (*SD* = 12.6 years). The gender distribution was quite balanced (48.9% males, 51.1% females). The majority had attained a university or college education (72.1%). Characteristics of the study sample are presented in Table 1.

**Table 1:** Characteristics of the study sample (*N* = 779).

**Variable**	
Age, *M* (*SD*)	40.3 (12.6)
**Gender**	
Male, *n* (%)	381 (48.9)
Female, *n* (%)	398 (51.1)
**Educational attainment**	
Primary/lower secondary, *n* (%)	217 (27.9)
University/college, *n* (%)	562 (72.1)
**Job position**	
Worker, *n* (%)	658 (84.5)
Manager, *n* (%)	121 (15.5)
**Work division**	
Transportation/manufacturing, *n* (%)	93 (11.9)
Public administration/services, *n* (%)	581 (74.6)
Health services, *n* (%)	92 (11.8)
Other services, *n* (%)	13 (1.7)

### Measures

In line with earlier studies on willingness to participate in alcohol prevention interventions (35–38), a set of sociodemographic and alcohol-related variables was included as potential correlates. Additionally, work-related variables were included due to the target population being employed and that alcohol prevention interventions were delivered in an occupational health setting. A set of lifestyle/daily activity variables was included as a result of the potential importance of contextual factors outside the workplace. An overview of included potential correlates is presented in Table 2.

**Table 2:** Unadjusted associations with willingness to participate.

**Variable**	**Willing**	**Unwilling**	**Difference (*p value*)**
**Sociodemographic factors**			
Age, *M* (*SD*)^†^	41.4 (12.4)	39.7 (12.6)	0.059^a^
Gender, % females	48.8	52.5	0.320^b^
Educational attainment, % university/college	72.1	72.2	0.965^b^
Marital status, % married	37.0	34.6	0.499^b^
Living status, % living with others	74.7	75.5	0.809^b^
Having children, % yes	58.9	56.2	0.460^b^
Having children in household, % yes	40.1	40.7	0.869^b^
Yearly household income^c^, *M* (*SD*)	918.9 (423.2)	933.8 (718.9)	0.754^a^
**Alcohol-related factors**			
Alcohol use and consequences, *M* (*SD*)	10.3 (2.8)	10.4 (2.9)	0.541^a^
Drinking attitudes, *M* (*SD*)^†^	2.5 (0.4)	2.4 (0.5)	0.087^a^
Alcohol expectancies, *M* (*SD*)	2.2 (0.5)	2.2 (0.5)	0.902^a^
Alcohol-related presenteeism, *M* (*SD*)^†^	0.2 (0.7)	0.3 (0.8)	0.066^a^
Alcohol-related impaired activities, *M* (*SD*)	0.6 (1.2)	0.6 (1.2)	0.674^a^
**Work-related factors**			
Job size, *M* (*SD*)^*^^†^	95.5 (16.9)	91.9 (20.9)	0.010^a^
Job position, % worker^***^^†^	78.1	88.4	<0.001^b^
Typical work hours per day, *M* (*SD*)^†^	8.0 (1.8)	7.7 (2.2)	0.110^a^
Psychological job demands, *M* (*SD*)	2.6 (0.5)	2.6 (0.5)	0.679^a^
Workplace decision latitude, *M* (*SD*)^†^	3.0 (0.4)	3.0 (0.4)	0.154^a^
Workplace social support, *M* (*SD*)	3.1 (0.5)	3.1 (0.5)	0.392^a^
Work effort, *M* (*SD*)^†^	2.9 (0.6)	2.8 (0.6)	0.207^a^
Work reward, *M* (*SD*)	2.6 (0.4)	2.6 (0.4)	0.952^a^
Effort-reward imbalance ratio, *M* (*SD*)^†^	2.6 (0.9)	2.6 (0.8)	0.298^a^
Work overcommitment, *M* (*SD*)^†^	2.3 (0.6)	2.4 (0.6)	0.210^a^
Employment sector^***^^†^			<0.001^b^
Private	19.2	11.2	
Local government	51.2	64.9	
Central government	29.6	23.9	
Work division^**^^†^			0.005^b^
Transportation/Manufacturing, %	17.2	8.7	
Public administration/services, %	69.7	77.6	
Health services, %	11.8	11.8	
Other services, %	1.3	1.9	
**Lifestyle/daily activity factors**			
Sleep/rest, *M* (*SD*)	7.7 (1.5)	7.7 (1.6)	0.856^a^
Housework, *M* (*SD*)	1.5 (1.0)	1.6 (1.0)	0.586^a^
Care activities, *M* (*SD*)*^†^	1.4 (1.0)	1.6 (1.2)	0.027^a^
Media activities, *M* (*SD*)	3.0 (1.6)	3.0 (1.6)	0.976^a^
Culture activities, *M* (*SD*)	0.8 (0.8)	0.8 (0.8)	0.777^a^
Physical activity, *M* (*SD*)	1.7 (1.2)	1.6 (1.4)	0.708^a^

*Results from unadjusted bivariate analyses; M, mean; SD, standard deviation*.

a *Difference tested with independent samples t-test*.

b *Difference tested with chi square test of independence*.

c *In 1000 Norwegian kroner (NOK)*.

* *p < 0.05*;

** *p < 0.01*;

*** *p < 0.001*.

† *Variable eligible for inclusion in adjusted analysis due to p < 0.30*.

#### Willingness to Participate (Outcome)

Employees were coded as either willing (1) or unwilling (0) to participate in an alcohol prevention intervention. Willing employees fulfilled the following criteria: (i) responded positively to the invitation from the OHS; (ii) attended the health examination; and (iii) agreed to be randomized into an alcohol prevention or control condition.

#### Sociodemographic Variables

The following sociodemographic variables were measured: *Age* (years); *gender* (male; female); *educational attainment* (primary/secondary; university/college); *marital status* (unmarried; married); *living status* (living alone; living with others); *having children* (yes; no), *having children in the household* (yes; no); and *yearly household income* [in 1000 Norwegian kroner (NOK)].

#### Alcohol-Related Variables

*Alcohol use and consequences* were measured with the 10-item Alcohol Use Disorders Identification Test (AUDIT) (7, 43). Each item was scored 0–4, resulting in a sum score ranging from 0 to 40 (higher score indicated higher consumption/more severe consequences). *Drinking attitudes* were measured with seven items from the Drinking Norms Scale (DNS) (53). Each item was scored on a four-point Likert scale, and a mean score was calculated (higher score indicated more liberal attitudes toward drinking in general and work-related drinking in particular). *Alcohol expectancies* were measured with the 8-item short version of the Alcohol Expectancy Questionnaire (AEQ) (54). Each item scored on a four-point Likert scale, and a mean score was calculated (higher score indicated more positive expectancies). *Alcohol-related presenteeism*, i.e., decreased on-the-job performance associated with alcohol consumption, was measured with a single item from the Work Productivity and Activity Impairment questionnaire (WPAI) (55). On a visual analog scale ranging from 0 (no influence on productivity) to 10 (obstructed productivity completely), respondents answered the following question: “During the past 7 days, how much did your alcohol consumption affect your productivity while you were working?.” *Alcohol-related impaired daily activities* were similarly measured with a single WPAI-item: “During the past 7 days, how much did you alcohol consumption affect your ability to do your regular activities, other than work at a job?.”

#### Work-Related Variables

*Job size* was measured as percentage of full-time work, and *typical work hours per day* were measured by asking respondents to report how many hours they worked (at or outside the workplace) on a typical day. *Job position* (worker; manager); *employment sector* (private; local government; central government) and *work division* (transportation/manufacturing; public administration/services; health services; other services) were measured with categories. *Psychological job demands* were measured with five items from the Job Content Questionnaire (JCQ) (56), each scored on a four-point Likert scale. A mean score was calculated (higher score indicated higher demands). Similarly, *workplace decision latitude* (employees' perceived control over workplace decisions and perceived possibility of utilizing personal skills in the job) and *workplace social support* (from managers and co-workers) were measured with items from the JCQ scored on four-point Likert scales. Mean scores were calculated (decision latitude: nine items; social support: eight items). Higher scores indicated more perceived control and more social support. A short version of the Effort-Reward Imbalance questionnaire (ERI) (57, 58) was used to measure *work effort* (mean score of three items; higher score indicated higher effort), *work reward* (mean score of seven items; higher score indicated higher reward) and *work overcommitment* (exorbitant work ambition and need for approval; mean score of six items, higher score indicated higher overcommitment). All ERI-items were scored on four-point Likert scales. An *effort-reward imbalance ratio* was calculated with the following formula (57):

$$\begin{array}{l} \frac{M\;score\;effort}{M\;score\;reward\;\times \left(\frac{n\;effort\;items}{n\;reward\;items}\right)} \end{array}$$

#### Lifestyle/Daily Activity Variables

Respondents were asked to indicate how many hours they typically spent per day on the following activities: *media activities* (e.g., television, computer, internet); *culture activities* (e.g., concerts, restaurant and café visits, cinema, religious, and cultural ceremonies); *physical activity* (moderate and vigorous intensity exercise); *sleep/rest*; *housework*; and *care activities* (caring for oneself and/or others).

### Analysis

The proportion (percentage) of risky drinkers who was willing to participate in an alcohol prevention intervention was assessed. Correlates for willingness to participate were analyzed by comparing those who were willing to participate with those who were eligible but unwilling to participate, based on sociodemographic, alcohol-related, work-related, and lifestyle/daily activity variables (see Table 2). First, a series of unadjusted analyses was conducted (independent samples *t*-tests with Cohen's *d* for continuous correlates, and chi square tests of independence with phi coefficients (ϕ; 2 × 2 tables), or Cramer's V (larger tables) for categorical correlates). Second, correlates demonstrating associations with willingness to participate at *p* < 0.30 were included in an adjusted binary logistic regression analysis (59). All analyses were performed with IBM SPSS version 27. Significant results were defined as *p* < 0.05.

### Ethics

Respondents were treated in accordance with the World Medical Association's Declaration of Helsinki (60), and systematic efforts were made to ensure their dignity, integrity, right to self-determination, privacy, and confidentiality. At three time points (screening, invitation from the OHS, and health examination), participants were thoroughly informed about the study's aim and assured that participation was voluntary. Participants were informed that they had the right to withdraw from the study at any given time. Written informed consent was collected from all participants. The study was approved by the Regional Committee for Medical and Health Research in Norway (REK; reference number 2014/647).

## Results

### Willingness to Participate

Of 779 eligible risky drinking employees, 297 (38.1%) were willing to participate in an alcohol prevention intervention, while 481 (61.9%) were not.

### Correlates for Willingness to Participate

A series of unadjusted analyses comparing characteristics of willing and unwilling employees revealed that 5 out of a total of 31 variables demonstrated significant associations with willingness to participate (see Table 2).

Willing employees had a somewhat higher percentage of full-time work compared to their unwilling counterparts [*M*_diff._ = 3.6 percentage points, *t*_(777)_ = −2.57, *p* = 0.010, Cohen's *d* = −18], and managers were more willing than workers to participate [$\chi_{\left(1,\text{n}=779\right)}^{2}$ = 14.77, *p* < 0.001, ϕ = 0.14]. Employees employed by private companies and central government tended to favor participation more than those employed by local governments [$\chi_{\left(2,\text{n}=779\right)}^{2}$ = 16.41, *p* < 0.001, Cramer's V = 0.15]. Employees in transportation/manufacturing tended to favor participation, while employees in public administration/services tended to favor non-participation [$\chi_{\left(3,\text{n}=779\right)}^{2}$ = 12.85, *p* = 0.005, Cramer's V = 0.13]. Employees who spent less time on care activities were somewhat more prone to participate than those who spent more time on care activities [*M*_diff._ = 0.2 h per day, *t*_(777)_ = 2.35, *p* = 0.019, Cohen's *d* = 0.16]. According to Cohen's guidelines (61), all these significant correlates were characterized by small effect sizes. None of the sociodemographic or alcohol-related variables demonstrated bivariate significant associations with willingness to participate. However, the following correlates were deemed eligible for inclusion in an adjusted multiple analysis due to *p*-values lower than 0.30: age (*p* = 0.059), drinking attitudes (*p* = 0.087), alcohol-related presenteeism (*p* = 0.066), typical work hours per day (*p* = 0.110), workplace decision latitude (*p* = 0.154), work effort (*p* = 0.207), effort-reward imbalance (*p* = 0.298), and work overcommitment (*p* = 0.210).

The adjusted analysis contained 13 correlates, was statistically significant [$\chi_{\left(13,\text{n}=779\right)}^{2}$ = 48.72, *p* < 0.001] and explained between 6.1% (Cox & Snell *R*^2^) and 8.3% (Nagelkerke *R*^2^) of the variance in willingness to participate (see Table 3).

**Table 3:** Adjusted associations with willingness to participate.

			**95% CI for OR**	
**Variable**	**OR**	***p***	**Lower**	**Upper**
Age	1.01	0.400	0.99	1.02
Drinking attitudes	1.43	0.058	0.99	2.06
Alcohol-related presenteeism	0.78^*^	0.028	0.62	0.97
Job size	1.01	0.072	1.00	1.02
Job position^a^	2.17^**^	0.001	1.40	3.35
Typical work hours per day	1.01	0.881	0.93	1.09
Employment sector	1.01	0.947	0.75	1.36
Workplace decision latitude	0.62^*^	0.020	0.42	0.93
Work effort	1.19	0.456	0.75	1.89
Effort-reward imbalance ratio	1.09	0.584	0.80	1.50
Work over-commitment	1.49^*^	0.011	1.10	2.04
Work division	1.22	0.253	0.86	1.72
Care activities	0.84^*^	0.018	0.72	0.97

*Results from multiple binary logistic regression*.

*Cox and Snell R^2^ = 0.061; Nagelkerke R^2^ = 0.083*.

*OR, odds ratio; CI, confidence interval*.

a *ref, worker*;

* *p < 0.05*;

** *p < 0.01*.

Five correlates demonstrated significant associations with willingness to participate in the adjusted analysis. Being willing to participate was significantly associated with less alcohol-related presenteeism (OR = 0.78, *p* < 0.05, 95% CI: 0.62, 0.97), more work overcommitment (OR = 1.49, *p* < 0.05, 95% CI: 1.10, 2.04), less decision latitude (OR = 0.62, *p* < 0.05, 95% CI: 0.42, 0.93), and less time spent on care activities (OR = 0.84, *p* < 0.05, 95% CI: 0.72, 0.97). Managers were significantly more willing to participate than workers (OR = 2.17, *p* < 0.01, 95% CI: 1.40, 3.35).

**Figure 2:**
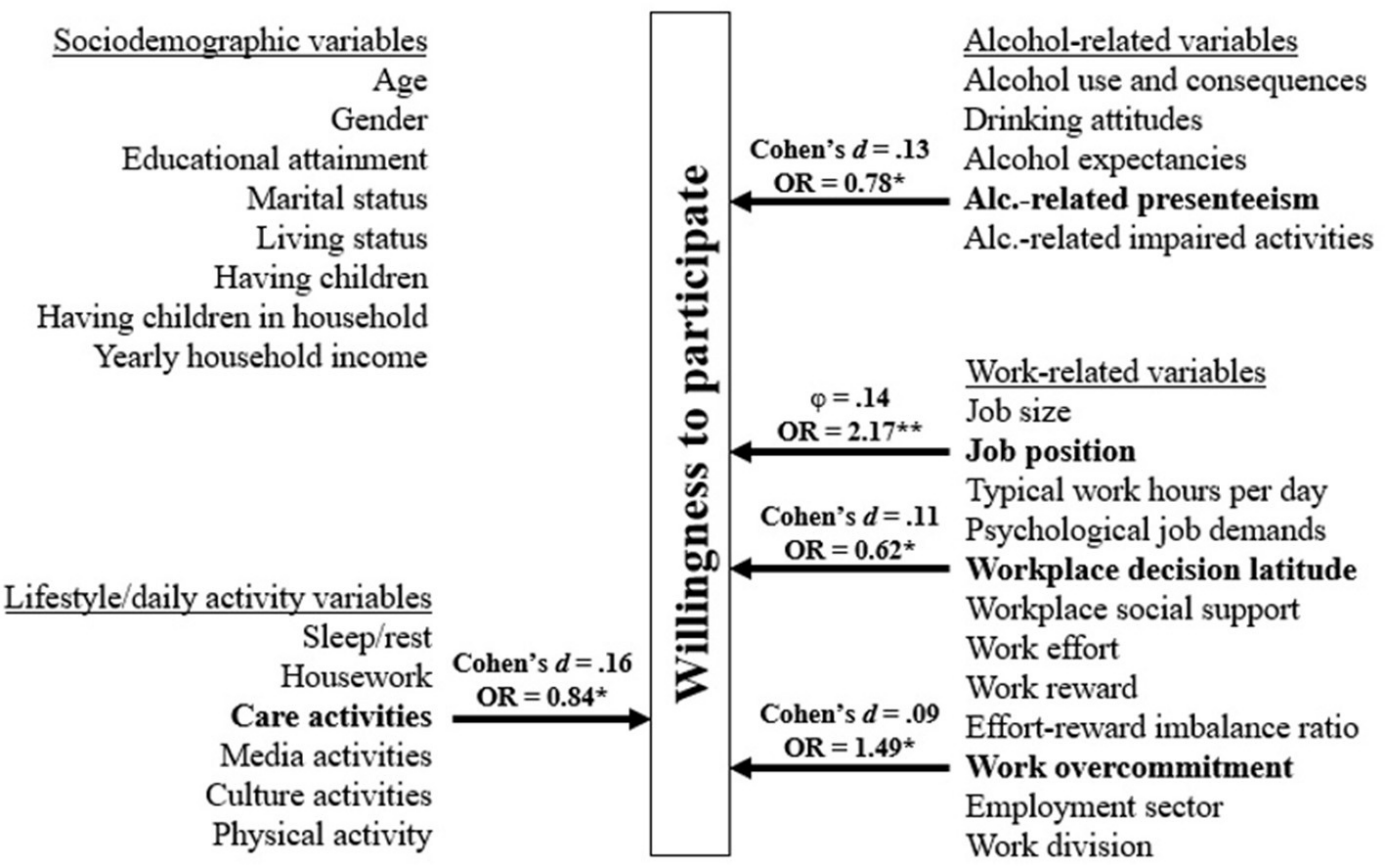
Overview of correlates' associations with willingness to participate in alcohol prevention interventions. The five correlates that demonstrated significant associations with willingness to participate in the adjusted analysis are presented in terms of both unadjusted and adjusted measures. ϕ = *phi* coefficient; *p < 0.05; **p < 0.01.

Figure 2 presents an overview of explored correlates' associations with willingness to participate. Overall, only 5 out of 31 correlates were significantly associated with willingness to participate, and these 5 associations were weak (ϕ and Cohen's *d* between 0.09 and 0.16).

Sensitivity analyses (see Supplementary Material) were performed in order to explore the extent to which results presented in Tables 2, 3 changed when adjusting for gender and age at each step. Sensitivity analyses showed that main results did not change substantially, even when adjusting for gender and age.

## Discussion

In accordance with the RE-AIM framework (29, 30), the extent to which a target population is reached for an intervention (i.e., the proportion of eligible individuals who is willing to participate, and how representative willing individuals are compared to the target population) is an important issue when evaluating the public health impact of interventions. Also, knowledge about reach in a particular context is pivotal for enabling better recruitment and implementation strategies for future intervention initiatives. This study, conducted in a heterogeneous sample of employees in Norway, aimed to estimate the proportion of risky drinkers who were willing to participate in an alcohol prevention intervention in an occupational health setting, and to explore correlates for such willingness. The results showed (i) that 38.1% of risky drinkers were willing to participate, and (ii) that willing employees were quite comparable to unwilling, based on exploration of 31 potential correlates.

Approximately 4 out of 10 eligible employees were willing to participate. This participation rate is considerably lower than rates demonstrated among trauma patients in the USA (60.8%; brief face-to-face intervention delivered by health care personnel) (37), regular drinkers in a German general population sample (67.0%; digital intervention with computer-generated feedback) (38), and risky drinking hospital inpatients in Germany (81.0%; digital intervention with computer-generated individualized feedback, and face-to-face intervention delivered by health care personnel) (36). Yet, the participation rate in our study is quite comparable with the rate found among emergency department patients in Sweden (41.0%; computerized intervention) which, according to the authors of the Swedish study, represented an acceptable reach (35). Interpretation of our results should take into account that being willing to participate, i.e., attending the health examination at OHS' facilities, did require notable practical efforts. Employees had to leave their workplace during working hours and in some cases travel some distance. A somewhat higher proportion of employees could have been willing to participate if necessary practical barriers were minimized, e.g., if employees were simply offered to participate directly in a digital intervention. Moreover, risky drinkers may be reluctant to perceive their alcohol use as problematic due to not yet having experienced any alcohol-related problems, which may have lowered their willingness to invest notable practical efforts to participate in an alcohol prevention intervention. Unlike patients, who are already in a treatment setting, employees should be considered a mainly non-clinical population, which may explain why the willingness to participate was considerably lower in our study compared to studies utilizing patient samples. In our study, it is unclear whether and how willingness to participate may have been affected by the fact that employees were offered to participate in an intervention as part of a research study.

Overall, risky drinking employees may constitute between 10 and 30% of the workforce (6). For the European Union with a workforce of 238.9 million workers (62), this translates into between 23.9 and 71.7 million individuals. Thus, reaching four out of ten risky drinking employees for prevention interventions may carry a considerable public health impact, even though our results imply that risky drinking employees are challenging to reach.

Our results indicate that employees willing and unwilling to participate were quite comparable on a large number of variables. Based on exploration of a total of 31 potential correlates (covering a wide range of sociodemographic, alcohol-related, work-related and lifestyle/daily activity variables), only 5 of these correlates were significantly associated with willingness to participate, and these 5 associations carried small effect sizes.

None of the sociodemographic variables (age, gender, educational attainment, marital status, living status, having children, having children in household, yearly household income) was significantly associated with willingness to participate. Some studies have found that younger individuals are more prone to participate in alcohol interventions than older individuals (35, 36, 38). However, we did not find such an association. In studies of hospital patients and visitors of a registry office in Germany (36, 38), individuals with higher education were found to be more willing to participate, compared to their lower education counterparts. However, and in line with a study of Swedish patients (35), we did not find any differences in willingness to participate in alcohol prevention interventions based on educational attainment. It should be kept in mind that more than seven out of ten employees in our sample had completed a university/college education.

Interestingly, variables such as alcohol use and consequences, drinking attitudes and alcohol expectancies did not predict willingness to participate. As such, one may assume that willing and unwilling employees were not systematically different with regard to how much they drank, how they construed drinking in general as well as work-related drinking more specifically, and what effects they expected alcohol to produce. These findings are in line with previous studies that have revealed no systematic differences in terms of intervention participation based on weekly consumption and frequency of heavy episodic drinking (35), overall alcohol use and consequences (36), typical number of drinks per week (38), or blood alcohol concentration (37). We did, however, find that those willing to participate reported less alcohol-related presenteeism, i.e., occurrences of episodes where on-the-job performance have been thwarted by alcohol consumption. This difference was very small and corresponded, on average, to 0.1 points on a scale ranging from 0 to 10.

Willingness to participate did not vary significantly between employment sectors or divisions when adjusting for other correlates. Moreover, willing and unwilling employees were not significantly different regarding job size, typical work hours, perceived psychological job demands, workplace social support, work effort, work reward or the balance between perceived effort and reward. We found, however, that managers were approximately twice as likely as workers to participate. Managers may, compared to workers, have felt a greater sense of obligation and a responsibility to set a good example, as well as being less inclined to worry about potential negative consequences of seeking and receiving help. This observed difference may also be due to managers being better informed than workers about the study and its procedures, and they may have found it easier to leave the workplace during work hours without being noticed. Unfortunately, our data cannot explain these observed differences. Employees willing to participate reported somewhat more work overcommitment and less control over workplace decisions (decision latitude) than those unwilling to participate. This may, to some extent, reflect that commitment and loyalty to the workplace, as well as experiencing rather limited flexibility and autonomy in the job, generate a willingness to participate in arrangements that are recommended by the employer. It should be noted that observed differences were marginal (on scales ranging from 1 to 4: 0.05 points for decision latitude, and 0.1 points for overcommitment).

Of the daily activity patterns measured (sleep/rest, housework, care activities, media activities, culture activities, physical activity), only time spent on care activities (caring for oneself and/or others) differed somewhat between employees who were willing and unwilling to participate. Willing employees spent 0.2 h (corresponding to 12 min) per day less on care activities than unwilling employees. In general, women are known to carry a larger burden of care activities than men (63). Interestingly, the association between time spent on care activities and willingness to participate did not change substantially when adjusting for gender and age (see Supplementary Material and Table 1 of Supplementary Material). One explanation may be that those having less care responsibilities perceived to have greater flexibility and more time to spend on participating in an alcohol prevention intervention. However, our data do not allow for conclusions regarding reasons for the observed differences.

### Methodological Considerations

This study has several strengths. First, a wide range of potential correlates were included (covering sociodemographic, alcohol-related, work-related, and lifestyle/daily activity factors), for the most part measured with validated instruments, e.g., the Alcohol Use Disorders Identification Test (AUDIT) (7, 43), the Drinking Norms Scale (DNS) (53), the Alcohol Expectancy Questionnaire (AEQ) (54), the Job Content Questionnaire (JCQ) (56), and the Effort-Reward Imbalance questionnaire (ERI) (57, 58). Second, although we did not measure whether employees actually participated in an intervention, we did measure their willingness to do so and whether they made notable practical efforts (leave the workplace and attend a health examination at OHS' facilities) in order to be allocated into an intervention (or control condition). As such, our outcome did not solely rely on surveying how employees would behave *if* they had been offered an intervention. Third, our sample comprised a relatively large number of employees (*N* = 779) across sectors and work divisions, and we were able to demonstrate that willingness to participate did not differ significantly according to such variables.

On the other hand, certain limitations should be kept in mind when interpreting results from this study. Our sample comprised risky drinkers who initially were willing to participate in an alcohol screening survey. The proportion of risky drinkers among employees unwilling to participate in the initial screening is unknown. Moreover, we do not know how many of these risky drinking non-respondents would have agreed to participate in an alcohol prevention intervention had they been offered one. Therefore, our findings may not be directly applicable to risky drinkers in the workforce as a superordinate population.

Drinking and work cultures may vary across countries. This study was conducted within a Norwegian context and, as such, within a Norwegian drinking and work culture. This may limit the generalizability of our findings. Moreover, selection bias may to some extent have been inherent in this study. Although the sample distribution of males and females was similar to the distribution in the Norwegian workforce (females: sample = 51.1%; workforce = 47.3%) (64), employees with a university/college education were clearly overrepresented in our study. According to Statistics Norway (64), 41.1% of the workforce have attained university/college education, while the corresponding proportion in the sample was 72.1%. However, the study sample was more similar to the population of public sector employees in Norway (72.7% with university/college education), which may be due to employees in public administration/services constituting 74.6% of the study sample.

Our adjusted model, containing 13 potential correlates (who had demonstrated bivarate associations with willingness to participate at *p* < 0.30) accounted for a very small amount of variation in the outcome (between 6.1 and 8.3%). This indicates that choices of whether or not to take part in an alcohol prevention intervention could be largely explained by variables beyond those measured in this study. The extent to which participation required notable practical efforts may have been important. Also, opting to participate may, to a considerable extent, be explained by idiosyncratic variables that are difficult to measure. Future research could benefit from including individual-level variables, such as personality traits, mental health and well-being, motivation for change, and attitudes toward help-seeking behavior when exploring willingness to participate in prevention interventions. This study does neither illuminate proportion of employees who adhered to the interventions, nor whether adherence was dissimilar for different types of interventions (face-to-face vs. digital interventions). In terms of the RE-AIM framework, our study focused on one aspect of reach, i.e., the willingness to participate rather than actual participation rate. Retention and drop out constitute avenues for future research. Lastly, it should be kept in mind that employees were invited to participate in interventions as part of a randomized controlled trial, not as part of routine OHS practice. It is unclear whether and to what extent this may have influenced willingness to participate.

### Implications

This study implies that employed risky drinkers constitute a challenging, yet not unreachable, population for alcohol prevention in occupational health settings, and that employees willing to participate in such interventions are fairly similar to those who are not, based on the variables measured in this study. However, our findings imply that recruitment and implementation strategies should take certain work-related variables into account. Workers were less willing to participate than managers, suggesting the importance of ensuring secure commitment among workers. De-stigmatizing alcohol prevention interventions and assuring workers about anonymity and data security may be serviceable. Less than half of the eligible risky drinkers were willing to participate, and risky drinkers may not perceive their alcohol use as problematic due to a lack of experienced alcohol-related problems. Hence, primary prevention efforts, e.g., alcohol education with an emphasis on risk knowledge, may be expedient. Studies have shown that primary workplace-based interventions can improve risk knowledge (65) and improve motivation for consumption reduction (66). Regardless of the similarities between those willing and unwilling to participate in an alcohol-related prevention intervention, increasing willingness is a goal in itself to ensure public health impact. Further research on barriers against participating in alcohol prevention interventions among employees is warranted, by means of both quantitative and qualitative research designs.

## Conclusions

In this study, 4 out of 10 risky drinking employees were willing to participate in alcohol prevention interventions in an occupational health setting. Willing and unwilling employees differed significantly on 5 out of 31 variables of interest. Hence, employees with a risky drinking pattern may be somewhat challenging to reach for alcohol prevention interventions, and there seems to be few systematic differences between those who are willing and unwilling to participate in such interventions. The study strengthens the rationale for targeting risky drinking in workforce populations, and for conducting further research on barriers against participating in alcohol prevention interventions in occupational health settings.

## Data Availability Statement

Data from this study are available from the project owner (University of Stavanger, Faculty of Health Sciences, Department of Public Health) by principal investigator and project manager RA on reasonable request.

## Ethics Statement

This study, involving human participants, was reviewed and approved by Regional Committee for Medical and Health Research in Norway (REK; Reference No. 2014/647). The patients/participants provided their written informed consent to participate in this study.

## Author Contributions

RA is the principal investigator (PI) and project manager (PM) for the WIRUS project (Workplace Interventions preventing Risky alcohol Use and Sick leave). This study was designed by MT and RA. MT analyzed the data and drafted the manuscript. TB, JS, LS, AS, WM, and RA provided scientific input to the different drafts and provided data interpretation. All authors made critical revisions and provided intellectual content to the manuscript, approved the final version to be published, and agreed to be accountable for all aspects of this work.

## Conflict of Interest

WM wishes to declare that he also is director of Evalua Nederland B. V. (www.evalua.nl) and non-executive board member of Arbo Unie B. V. (www.arbounie.nl). Both companies are active in the Dutch occupational health care market. The remaining authors declare that the research was conducted in the absence of any commercial or financial relationships that could be construed as a potential conflict of interest.
