# Supplementary material for: Willingness to Participate in Alcohol Prevention Interventions Targeting Risky Drinking Employees. The WIRUS Project
Source: Front Public Health. 2021 Jun 25;9:692605. doi: 10.3389/fpubh.2021.692605 (PMC8267363; doi:10.3389/fpubh.2021.692605)
Supplement: Supplementary file 1 [file Table_1.DOCX]

Supplementary Material

# Sensitivity analyses

Sensitivity analyses were performed in order to explore whether and the extent to which unadjusted associations between correlates and the outcome changed when adjusting for gender and age. First, each unadjusted association presented in the article’s Table 2 was re-analyzed by including gender and age as covariates in multiple binary logistic regression analyses (see Table S1).

| **Table S1.** Associations between predictors and the outcome (willingness to participate), adjusted for gender and age | | |
| --- | --- | --- |
| Variable | OR | *p*-value |
| **Sociodemographic factors** |  |  |
| Age | 1.01^A^ | .078^A^ |
| Gender | 1.11^B^ | .457^B^ |
| Educational attainment | 1.05 | .990 |
| Marital status | 1.05 | .795 |
| Living status | 1.09 | .619 |
| Having children | 1.09 | .669 |
| Having children in household | 1.08 | .653 |
| Yearly household income | 1.00 | .589 |
| **Alcohol-related factors** |  |  |
| Alcohol use and consequences | 1.01 | .596 |
| Drinking attitudes | 1.44 | .049 |
| Alcohol expectancies | 1.02 | .936 |
| Alcohol-related presenteeism | 0.82 | .081 |
| Alcohol-related impaired activities | 1.03 | .620 |
| **Work-related factors** |  |  |
| Job size | 1.01 | .037 |
| Job position | 2.01 | .001 |
| Typical work hours per day | 1.05 | .174 |
| Psychological job demands | 1.09 | .334 |
| Workplace decision latitude | 0.77 | .136 |
| Workplace social support | 1.08 | .625 |
| Work effort | 1.16 | .237 |
| Work reward | 1.01 | .962 |
| Effort-reward imbalance ratio | 1.09 | .306 |
| Work overcommitment | 1.18 | .205 |
| Employment sector | 1.04 | .727 |
| Work division | 1.35 | .042 |
| **Lifestyle/daily activity factors** |  |  |
| Sleep/rest | 1.01 | .824 |
| Housework | 1.04 | .586 |
| Care activities | 0.86 | .036 |
| Media activities | 1.00 | .930 |
| Culture activities | 1.02 | .807 |
| Physical activity | 1.03 | .597 |
| Results from multiple binary logistic regression analyses; OR = odds ratio; ^A^Adjusted for gender; ^B^Adjusted for age | | |

Drinking attitudes reached statistical significance when adjusting for gender and age (from *p* = .087 to *p* = .049). However, this correlate was already included in the adjusted analysis presented in the article’s Table 3 due to having an initial *p*-value lower than .30. Effort-reward imbalance ratio remained non-significant, but the *p*-value increased from .298 to .306. Employment sector lost its statistical significance (from *p* <.001 to *p* = .727). Hence, these two correlates (effort-reward imbalance ratio and employment sector) could have been excluded from the adjusted analysis presented in the article’s Table 3 due to having *p*-values higher than .30 when adjusted for gender and age.

Second, the adjusted analysis in the article’s Table 3 was re-run without effort-reward imbalance ratio and employment sector as correlates (see Table S2).

| **Table S2.** Adjusted associations with willingness to participate | | | | |
| --- | --- | --- | --- | --- |
|  |  |  | 95 % CI for OR | |
| Variable | OR | *p* value | Lower | Upper |
| Age | 1.01 | .403 | 0.99 | 1.02 |
| Drinking attitudes | 1.42 | .060 | 0.99 | 2.06 |
| Alcohol-related presenteeism | 0.78 | .029 | 0.62 | 0.98 |
| Job size | 1.01 | .067 | 1.00 | 1.02 |
| Job position^a^ | 2.13 | .001 | 1.38 | 3.28 |
| Typical work hours per day | 1.01 | .849 | 0.93 | 1.10 |
| Workplace decision latitude | 0.60 | .008 | 0.41 | 0.88 |
| Work effort | 1.31 | .075 | 0.97 | 1.78 |
| Work overcommitment | 1.47 | .012 | 1.09 | 2.00 |
| Work division | 0.82 | .178 | 0.62 | 1.09 |
| Care activities | 0.84 | .021 | 0.72 | 0.97 |
| Results from multiple binary logistic regression; Cox and Snell *R*^2^ = .061; Nagelkerke *R*^2^ = .082; OR = odds ratio; CI = confidence interval; ^a^ref = worker | | | | |

The results presented in Table S2 do not deviate substantially from the initial results presented in the article’s Table 3: (i) the same correlates reached statistical significance (alcohol-related presenteeism, job position, workplace decision latitude, work overcommitment, care activities), (ii) effect sizes for these significant correlates changed only marginally (job position = 1.8 %; workplace decision latitude = 3.2 %; work overcommitment = 1.3 %; alcohol-related presenteeism = 0.0 %; care activities = 0.0 %), and (iii) the adjusted model’s proportion of explained variance in the outcome was nearly identical (from 6.1/8.3 % to 6.1/8.2 %).
